# Moisture Loss from Cheese During Baking: Influence of Cheese Type, Cheese Mass, and Temperature

**DOI:** 10.3390/foods14020165

**Published:** 2025-01-08

**Authors:** Justyna Tarapata, Ewa Szymańska, Liesbeth van der Meulen, Joost Miltenburg, Thom Huppertz

**Affiliations:** 1 Department of Dairy Science and Quality Management, Faculty of Food Science, University of Warmia and Mazury in Olsztyn, Oczapowskiego 7, 10-719 Olsztyn, Poland; justyna.tarapata@uwm.edu.pl; 2 FrieslandCampina, 3800LE Amersfoort, The Netherlands; ewa.szymanska@frieslandcampina.com (E.S.); liesbeth.vandermeulen@frieslandcampina.com (L.v.d.M.); joost.miltenburg@frieslandcampina.com (J.M.); 3 Food Quality & Design Group, Wageningen University and Research, 6708WG Wageningen, The Netherlands; 4 School of Food and Nutritional Sciences, University College Cork, T12 Y337 Cork, Ireland

**Keywords:** cheese baking, moisture loss, temperature, mass, initial moisture content, browning, mixed-effects model

## Abstract

This study examined how temperature, cheese mass and moisture content impact moisture loss rate in various cheeses during baking. Understanding these factors is essential for determining the browning properties of cheese during baking. Eight cheese types, differing in moisture content, were baked at 100–200 °C in a halogen moisture analyzer, and moisture loss over time was recorded. A mixed-effects model analysis showed that temperature had the most significant impact on moisture loss rate (F = 2008.54; *p* < 0.00001), followed by cheese mass (F = 1973.28; *p* < 0.00001) and time (F = 278.49; *p* < 0.00001). Higher temperatures and larger cheese mass accelerated moisture evaporation rate. The moisture content of cheese explained 21.8% of model variation, suggesting that other factors, such as cheese structure, also play a significant role. The cheese baking process involving moisture removal followed by distinct drying stages. Initially, the drying rate increased as the cheese reached the wet bulb temperature; this was followed by a steady rate, and finally, a reduction in dehydration rate as the moisture decreased, limiting further evaporation. Browning, assessed through changes in lightness (L*), was more noticeable at higher temperatures, particularly in Mozzarella and processed Cheddar, after approx. 30% moisture loss in all cheeses.

## 1. Introduction

Cheese is a versatile ingredient incorporated into a wide range of baked food products, i.e., pizza, burgers and cheesy baked pasta dishes, like lasagna or macaroni and cheese [1,2]. It is characterized by complex physical and chemical properties, which are shaped by its unique composition that change over ripening [3,4]. These properties are also fundamental to the behavior of cheese during baking, where moisture loss, texture changes, and browning occur [5,6]. Understanding these factors is essential not only for culinary applications, but also for industrial purposes, where precise control over moisture loss and browning is required to ensure product quality and stability.

The rate of moisture loss in cheese products during baking, along with its browning characteristics, is influenced by numerous factors, including baking temperature, initial moisture and fat content, and cheese mass [7]. Low-fat cheese usually exhibits poor melting properties. Increasing moisture content in low-fat cheese leads to improved meltability during baking [8]. Increasing the moisture-to-protein ratio in low-fat Mozzarella has also been found to influence its baking characteristics [9,10]. These changes were linked to reduced moisture loss during baking, helping to minimize excessive drying and browning and keeping the cheese surface adequately moist [2]. Studying these variables can elucidate the behavior of cheese during thermal treatment and offer insights for optimizing baking processes.

Moisture loss and browning are interrelated phenomena, as the reduction in moisture content can accelerate the Maillard reaction, which is responsible for browning in dairy products, as well as other food products [11]. Browning is a critical indicator of the extent of baking and is often used as a quality measure. The Maillard reaction is highly dependent on the amount and type of substrate available, i.e., reducing carbohydrates and free amino groups, and moisture content, as well as time-temperature combinations of the thermal treatment [12,13]. Acceptable levels of browning can enhance both the flavor and esthetic qualities of the product, contributing to a desirable golden-brown crust while retaining moisture in the core. Excessive browning can lead to over-crusting and burnt flavors, which are undesirable in many cheese applications [14]. Hence, monitoring the browning process and understanding its relation to moisture loss during baking in detail is important for optimizing baking conditions or optimizing cheese properties for specific baking conditions.

A computer vision system and image analysis have previously been used by researchers to study various quality parameters of pizza toppings [15,16] and the quantification of pizza baking properties [5,17]. Among the numerous methods used to quantify the kinetics of browning via color measurements and chemical analysis, the visual color change in food products has been successfully described using the CIELAB color indices [18,19]. Cheese whiteness (L*) is linked to its heterogeneous structure, which scatters light [20]. This measure is commonly used to track browning during baking or to assess color changes during ripening, as L* depends on the concentration of cheese components, which increases over ripening time due to moisture loss [21,22]. Furthermore, proteolysis, which occurs during ripening, results in the transformation of casein into a more soluble state and can cause a decrease in whiteness [21]. Factor a* is associated with the presence of reaction substrates [11]. In general, the color of cheese is a sensory attribute that can enhance the flavor perception of consumers. It is an important attribute that plays a vital role for the acceptance and marketing of cheese [20,23]. In studies on fat-free Mozzarella cheese [2], the onset of browning during baking was strongly associated with a measurable decrease in lightness (L* value) and increases in yellowness (b* value) and redness (a* value), highlighting the use of CIELAB color indices as a quantitative tool to monitor and characterize changes in color [2].

Given the importance of cheese in culinary applications, particularly in pizza, it is vital to understand how different cheese types respond to baking conditions. Baking conditions significantly affect the microstructure, composition, appearance, and flow properties of cheese [15,24]. Moreover, consumer preferences are influenced not only by the melting behavior and visual appeal of baked cheese, but also by the textural sensations perceived during consumption [25,26]. A number of cheeses are used as pizza cheese, i.e., natural Mozzarella, Cheddar, Provolone, Parmesan, Emmental, Romano, Ricotta, Mozzarella analog, processed cheese, and modified cheeses, as described by Ma et al. [5], but Gouda and Edam cheese are also used. The majority of research conducted to date relates to the baking properties of either Mozzarella or Cheddar cheese [6,20,27,28]. Furthermore, most of them have focused on functional properties of cheese, particularly those that influence consumer perception (i.e., browning, blistering, melting, oiling-off, stretching) [27,29]. Banville et al. [6] studied aged Mozzarella baked on a pizza model under standardized conditions (230 °C in a commercial forced-air oven for 210 s) mimicking commercial preparation methods. They observed that cheeses lost approximately 15% of their weight due to moisture evaporation during baking, with younger cheeses being more susceptible to dehydration. This process increased the solids content and altered the cheese’s physical and sensory characteristics. Heating caused structural changes in the cheese matrix, leading to the release of aqueous serum and fat, which contributed to its distinctive sensory attributes. In another study by Ma et al. [5], the baking performance (232 °C in convection oven for 5 min) of various cheeses on pizza was assessed, focusing on attributes like blistering and browning. The study quantified the color and its uniformity post-baking, alongside cheese properties. Factors such as elasticity, free oil, moisture content, water activity, and transition temperature were shown to influence color uniformity. For cheeses like Cheddar, Colby, and Edam, blisters did not form due to their lower elasticity. Meanwhile, Gruyere and Provolone exhibited less intense browning because the presence of free oil reduced moisture evaporation. Emmental showed minimal browning, primarily due to its low steam pressure [5]. Furthermore, Wang and Sun [16] examined the browning behavior of Cheddar and Mozzarella cheeses, the relationship between baking time and temperature, and the browning factor. The findings revealed distinct patterns for each cheese type. For Mozzarella cheese, the browning factor increased almost linearly with baking temperature when cooked for 2–4 min. In contrast, for Cheddar cheese, a linear increase in browning factor with temperature was observed between 70 and 130 °C after 8–12 min of heating. Above 130 °C, additional browning was minimal, likely due to the depletion of reducing sugars and amino groups required for the Maillard reaction. However, at temperatures exceeding 160 °C, Cheddar cheese exhibited excessive darkening due to scorching [16].

Despite these studies, there is a lack of studies investigating the fundamental mechanisms of water evaporation and the rate at which it occurs across different cheese types.

This study investigates the effects of temperature, initial moisture content, and cheese mass on moisture loss rate and browning in a range of cheese types during baking. By examining these parameters, this work aimed to enhance the understanding of cheese behavior during baking and contribute to the development of predictive models for the rate of moisture loss. The specific cheese types analyzed in this study include Mozzarella, Edam, Gouda, Cheddar, and processed cheese, each with distinct moisture levels, fat compositions, and different ripening stages. Browning was assessed using a custom developed software analysis of images of cheese.

## 2. Materials and Methods

**Figure 1:**
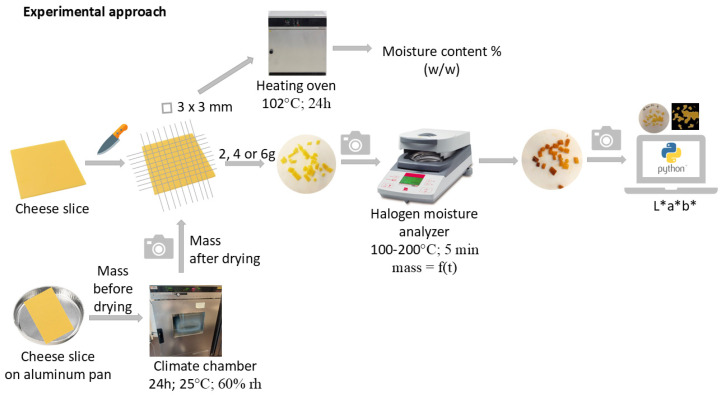
Experimental approach (rh = relative humidity).

The experimental approach used in this paper is displayed in Figure 1.

### 2.1. Materials

A block of Mozzarella, the balls of 16- and 40-week-old Edam, and sliced young, mature, and aged Gouda cheese, were provided by FrieslandCampina (Amersfoort, The Netherlands). Sliced processed Cheddar (Milbona, produced for Lidl Stiftung & Co. KG, Neckarsulm, Germany) and sliced mature Cheddar (Dubliner, Carbery Group, Ballineen, Ireland) were purchased from a retail store. The chemical composition of cheese, as claimed by the manufacturer, is provided in Table 1.

### 2.2. Methods

#### 2.2.1. Sample Preparation

All cheese samples were stored at 4 °C prior to use. The cheese blocks and balls were first sliced into 2 mm thick pieces using a slicing machine. These slices were then cut into 3 mm square pieces with a stainless-steel knife. The prepared samples were transferred onto glass fiber disks (88.9 mm; Mettler Toledo, Columbus, OH, USA) and an aluminum pan (VWR Scientific Products, Radnor, PA, USA), which was then placed in a halogen moisture analyzer (OHAUS MB Series; MB35; MB45, Greifensee, Switzerland).

#### 2.2.2. Moisture Content in Cheese Before Baking

The total solids content of cheese was determined using an oven drying method at 102 ± 2 °C (AOAC International, 2007; method 990.20; 33.2.44 [30]). The moisture content (M) was calculated as M (%) = 100 − total solids (%).

#### 2.2.3. Moisture Loss Rate During Baking of Cheese

The rate of moisture loss in the cheese samples was assessed to understand how temperature, cheese mass, and cheese type affect moisture loss over time (Figure 1). The samples of 2, 4, or 6 g of cheese, prepared as described in Section 2.2.1., were spread out on an aluminum pan lined with a glass fiber filter. The moisture loss rate was analyzed using the halogen moisture analyzer (OHAUS MB Series; MB35; MB45, Greifensee, Switzerland) set at temperatures of 100, 120, 140, 160, 180 or 200 °C for a 5 min test period. The residual weight was recorded every 20 s and used to calculate moisture loss.

Initially, the moisture content of each cheese sample was measured as described in Section 2.2.2. The halogen moisture analyzer was employed to measure the mass loss, which was then used to calculate the percentage of mass lost at each time point. The percentage of mass loss was equated to the percentage of moisture loss, assuming that the only component evaporating during baking was water. Using the initial moisture content and the percentage of mass loss over time, the moisture content of the cheese at each time point was calculated. This approach allowed for a time-based profile of moisture content during baking, providing insight into the kinetics of water evaporation for different cheese types under the applied baking conditions. The rate of moisture loss was calculated by dividing the total mass of moisture lost up to each time point during baking by the elapsed time from the beginning of baking to that point.

#### 2.2.4. Moisture Loss Rate During Baking of Pre-Dried Cheese

To evaluate the effect of initial moisture content of cheese on its baking properties, a pre-dried cheese was baked and the moisture content as a function of baking time was recorded. A slice of cheese was laid on the aluminum baking pan, which was 12 cm in diameter, and placed into a climate chamber (Memmert model, Schwabach, Germany) at 25 °C and 60% relative humidity for 24 h; this reduced the moisture content by approx. 30%. The moisture loss after 24 h was calculated from the difference in cheese mass measured before and after the incubation and the initial moisture content of the cheese. Dried cheese slices were cut into square pieces with side lengths of 2 mm using a stainless-steel knife, and a 2 g sample was subjected to baking tests as described in Section 2.2.3 in the temperature range from 100 to 160 °C.

#### 2.2.5. Image Acquisition and Color Analysis

To evaluate color changes in the cheese samples, digital images were captured before and after drying and after baking tests and saved in JPEG format for subsequent analysis. Image processing and color measurements (L*a*b* values) were conducted using a custom Python tool, primarily utilizing the OpenCV package.

In the processing workflow, the paper background in each image was first removed to isolate the cheese sample. Next, three representative colors were extracted from the image, excluding the black background. These colors were selected based on their ability to best represent the overall appearance of the sample. Each of these colors were then converted from RGB to the CIELAB color space, using D65 as the illuminant and a 2° observer angle. Finally, a weighted average color was calculated for both RGB and CIELAB values to represent each sample comprehensively.

The lightness value (L*) of the cheese samples was extracted from the obtained images. To assess browning, a browning factor (BF) was calculated using the method of Wang and Sun [16]. This factor, defined as the ratio of the lightness value before (L_0_*) and after (L_t_*) the simulated baking test, describes the browning property as follows:

BF = (L_0_*/L_t_*) × 100% (1)

Moreover, the total color change (ΔE), which represents the difference between the L*a*b* values of the cheese before and after baking, was calculated according to Esteller et al. [31] as follows:

ΔE = ((ΔL*)^2^ + (Δa*)^2^ + (Δb*)^2^)^1/2^ (2)

#### 2.2.6. Statistical Analysis

All the measurements were performed in duplicate. To investigate the effects of temperature, cheese mass, and initial moisture content, corresponding to the type of cheese, on the rate of moisture loss during cheese baking, a mixed-effects model was calculated using Minitab software (version 20.4, State College, PA, USA). The dependent variable was the rate of moisture loss. The fixed effects included time (measured at regular intervals during the baking process), temperature (baking temperature), and cheese mass (initial mass before baking). The random parameters were initial moisture content (moisture content of the cheese before baking) and replicate. The mixed-effects model was fitted using Restricted Maximum Likelihood (REML) estimation, and the significance of the fixed effects was assessed using Wald tests.

## 3. Results

The composition of the tested cheeses is shown in Table 1, highlighting the variation in moisture and fat content among different types. Mozzarella and processed Cheddar had the highest moisture content (46.6 and 56.0%, respectively), whereas aged Gouda, 40-weeks old Edam, and mature Cheddar exhibited a significantly (*p* < 0.05) lower moisture content (35.7, 36.1 and 36.3%, respectively).

**Figure 2:**
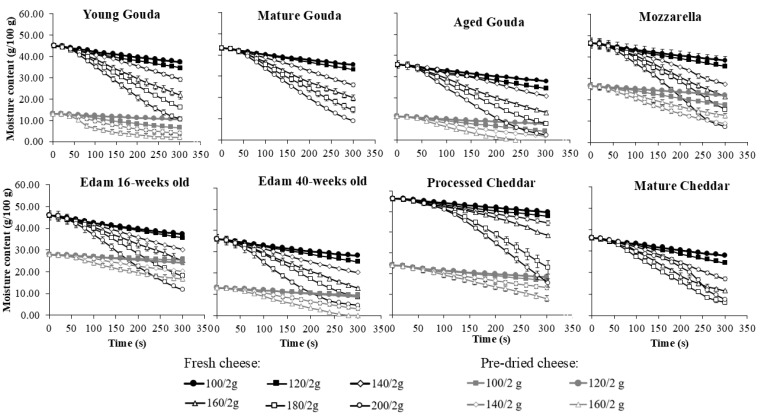
The effect of baking temperature and the initial moisture content of cheese on moisture content during baking of cheese (2 g) over time. Vertical error bars represent one standard deviation. For datapoints where error bars are not visible, the size of the error bar is smaller than the size of the symbol.

The moisture loss behavior of various cheeses (fresh and pre-dried) during baking was analyzed over 5 min of baking at different temperatures (100–200 °C) and for different sample masses (2, 4 and 6 g). The results show that moisture loss was influenced by cheese type, moisture content, ripening time, baking temperature, and cheese mass (Figure 2, Figures S1 and S2).

**Figure 3:**
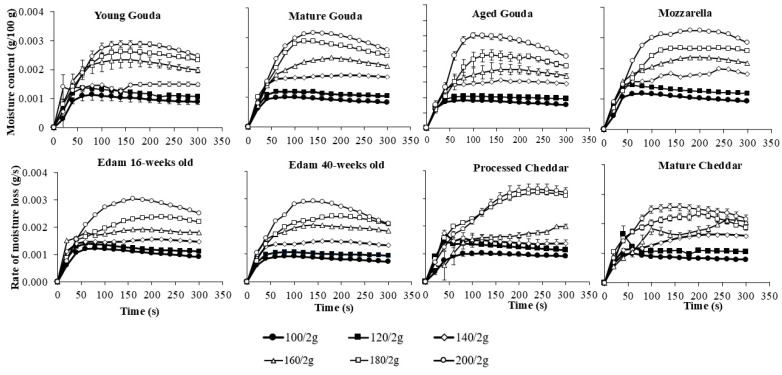
The effect of baking temperature on the rate of moisture loss in different cheese types (2 g). Vertical error bars represent one standard deviation. For datapoints where error bars are not visible, the size of the error bar is smaller than the size of the symbol.

The rate of moisture loss (Figure 3, Figures S3 and S4), which was calculated from changes in moisture contents as a function of time (Figure 2, Figures S1 and S2), generally followed a three-phase drying pattern: an initial rapid loss, followed by a steady rate phase, and finally a reduction in the rate of moisture loss as the cheese approached its final moisture level. The maximum moisture loss rate was reached faster at 100 and 120 °C (20–40 s for 2 g of cheese, and after ~50 s for 4 and 6 g) when compared to elevated temperatures (60–100 s for 2 g and 60–200 s for 4 and 6 g) and this depended on temperature and cheese type. The steady state was maintained either until the end of baking (5 min) or until the next phase, characterized by a decrease in the rate of moisture loss. The latter occurred for sample weights of 2 g for young, mature, and aged Gouda, Mozzarella, and mature Cheddar at temperatures ranging from 160 to 200 °C, as well as for Edam cheese (16- and 40-weeks old), processed Cheddar, and mature Cheddar at 180 to 200 °C (Figure 3). As the cheese mass increased to 6 g, the third phase (notable decrease in the moisture loss rate) was observed at elevated temperatures for certain cheese varieties. Specifically, this decrease was seen in mature and aged Gouda, 16- and 40-week-old Edam, and mature Cheddar at 200 °C (Figure S4). Moreover, at higher baking temperatures, the average rate of moisture loss increased rapidly across all cheese types, especially as temperatures rose from 140 to 200 °C (Figure 3). Furthermore, cheeses with higher initial moisture content, such as Mozzarella and processed Cheddar (Table 1), displayed faster rates of moisture loss compared to lower-moisture cheeses like mature Cheddar, Gouda, or Edam (Figure 3, Figures S3 and S4).

**Figure 4:**
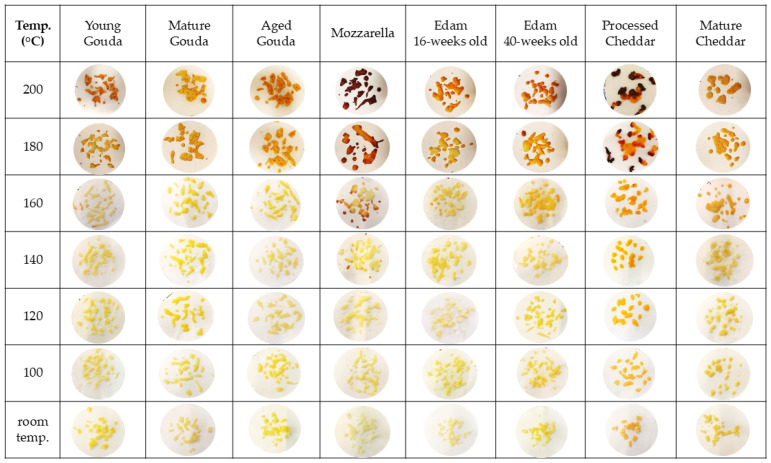
The effect of temperature on cheese (2 g) browning after 5 min of baking.

Cheese mass was also a significant factor, as larger cheese samples (4 and 6 g) exhibited a higher moisture loss rate compared to smaller samples (2 g) (Figure 3, Figures S3 and S4), suggesting that mass plays a role in escalating the rate of water transport to the surface. The increase in the rate of moisture loss was less than proportional to cheese mass, suggesting potential system-related effects, such as air circulation or evaporation capacity, on the observed rates. Reduced evaporation in larger samples is often due to overlapping cheese layers restricting moisture loss. In this study, all samples were cut to the same size, maintaining a consistent surface-to-weight ratio regardless of the cheese mass tested (Figure 4, Figures S5 and S6), eliminating the effect of overlapping.

Figure 4 illustrates how temperature affected browning in different types of cheese when 2 g of cheese sample was baked. As the temperature of baking increased the extent of browning also increased. In the case of Mozzarella and processed Cheddar, the first signs of browning were observed at 140 °C (Figure 4). Brown spots were visible on young Gouda, 16- and 40-week-old Edam, and mature Cheddar at 160 °C. In the case of mature and aged Gouda cheese, the onset of browning was observed at 180 °C. At 180 and 200 °C, processed Cheddar and Mozzarella were characterized by the darkest color and burned spots, compared to other cheese types, followed by cheese that undergone ripening (i.e., aged Gouda, 16- and 40-week-old Edam, and mature Cheddar). Moreover, the larger the mass of the baked cheese, the higher the temperature at which browning occurred (Figure 4, Figures S5 and S6). When 4 g of cheese was baked, the onset of browning was observed at 160 °C for Mozzarella, 40—weeks old Edam, and processed and mature Cheddar, while it was observed at 180 °C for other cheese types (Figure S5). However, when 6 g of cheese was baked, brown spots were first observed at 160 °C for mature Cheddar and 180 °C for the remaining cheese varieties (Figure S6).

**Figure 5:**
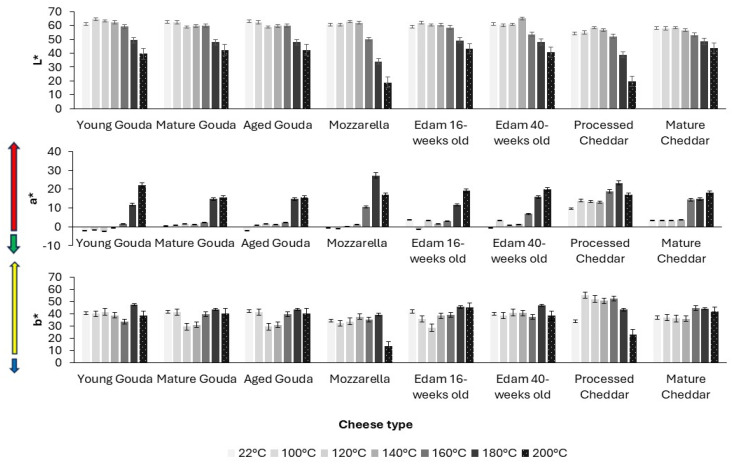
The effect of temperature on changes in color (CIELAB scale) of different types of cheese (2 g) after 5 min of baking. Vertical error bars represent one standard deviation.

**Figure 6:**
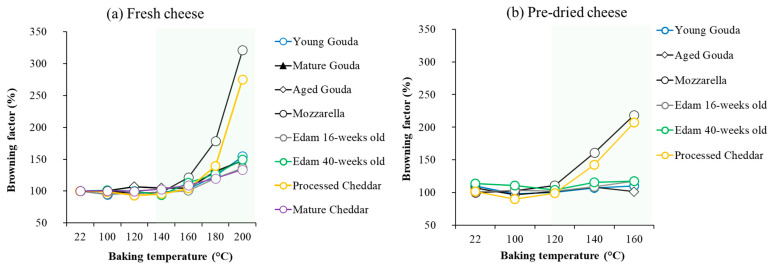
The browning factor calculated for (**a**)—fresh cheese samples (2 g) after 5 min of baking and (**b**)—pre-dried (24 h; 25 °C; 60% relative humidity) after 5 min of baking (2 g)**;** the green area represents the temperature range at which browning was observed visually.

The color of cheese has also been evaluated quantitatively. The color parameters, assessed by image analysis (Figure 5, Figures S7 and S8), corresponded to the visual observation (Figure 4) of the onset of browning as indicated by a reduction in L* (lightness) value and an increase in a* (redness) (Figure 5, Figures S7 and S8) above the temperature at which browning was visually observed (Figure 4, Figures S5 and S6). As the baking temperature increased, the extent of browning, measured by the change in L* value (lightness), also increased, with a more noticeable effect at higher temperatures (140–200 °C). The cheese samples started becoming significantly darker at 140–160 °C when compared to the unbaked control samples, for which lightness was measured at 22 °C (Figure 5 and Figure 6a). The browning factor, calculated as a ratio of lightness before and after baking, confirms this trend (Figure 6). The highest values of the browning factor were observed for Mozzarella and processed Cheddar when 2 g of cheese was analyzed (Figure 6). For 4 g, only Mozzarella was characterized by significantly higher browning compared to other cheeses within the tested baking temperature range (Figure S9). In general, the larger the cheese mass baked (2–6 g), the smaller the differences between browning factor calculated for all tested cheeses were observed (Figure 6 and Figure S9). When comparing the intensity of browning at 180 °C, where all cheeses exhibited visible brown spots, the a* redness value indicated that lower moisture content, resulting from cheese ripening, enhanced browning intensity (Figure 6). However, notable exceptions were Mozzarella and processed Cheddar cheese, which contained the highest moisture levels yet displayed the most intense browning. This suggests that initial moisture content alone does not fully explain browning. Additional factors, such as residual carbohydrates, like galactose and lactose in Mozzarella (0.7 and 0.1%, respectively) or carbohydrates in processed cheese, originating from ingredients like skim milk/skim milk powder (6 g/100 g; product label), likely contributed to browning through Maillard reactions during baking.

The total color difference (ΔE) was also calculated for fresh cheese samples, highlighting significant variations in browning intensity across different cheeses and baking temperatures (Table 2). For fresh cheese samples, the extent of color change (ΔE) remained relatively small at lower baking temperatures (100 and 120 °C) but increased at higher temperatures (180 and 200 °C). Among the samples, Edam (16 weeks old), Mozzarella, and processed Cheddar exhibited the highest ΔE values of 50.4, 49.9, and 35.6, respectively, after baking at 200 °C. Mature Cheddar demonstrated the least browning potential, with a ΔE value of only 21.4 at 200 °C. Gouda cheeses (young, mature, and aged) displayed increasing browning with rising temperature, reaching a ΔE of 32.4 (young Gouda), 25.2 (mature Gouda), and 27.1 (aged Gouda) at 200 °C.

**Figure 7:**
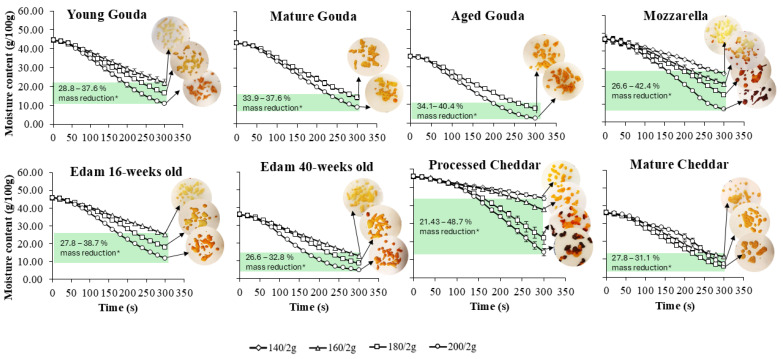
The effect of moisture loss during cheese baking (2 g) on the onset of browning. * The assumption is that, during baking, the cheese loses its mass only due to water evaporation. Vertical error bars represent one standard deviation. For datapoints where error bars are not visible, the size of the error bar is smaller than the size of the symbol.

Moreover, after comparing browning properties (Figure 4 and Figure 5) with the extent of moisture loss in cheese (Figure 2), it was observed that browning occurred when approx. 30% of moisture had evaporated from the cheese samples over the baking time (Figure 7).

**Figure 8:**
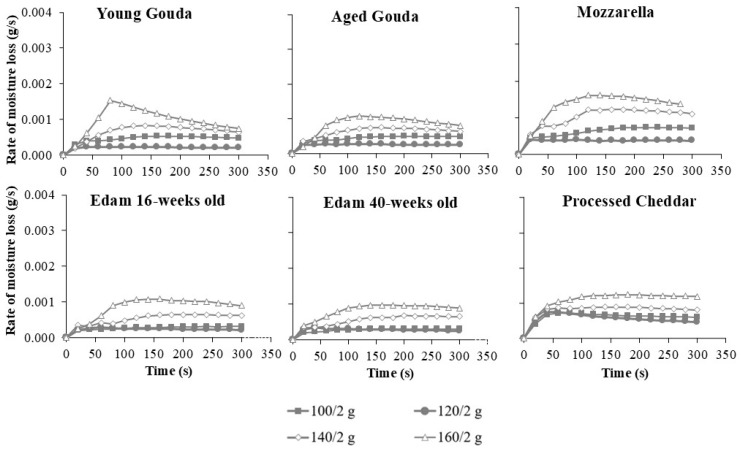
The effect of baking temperature on the rate of moisture loss in different pre-dried cheese types (2 g). Vertical error bars represent one standard deviation. For datapoints where error bars are not visible, the size of the error bar is smaller than the size of the symbol.

**Figure 9:**
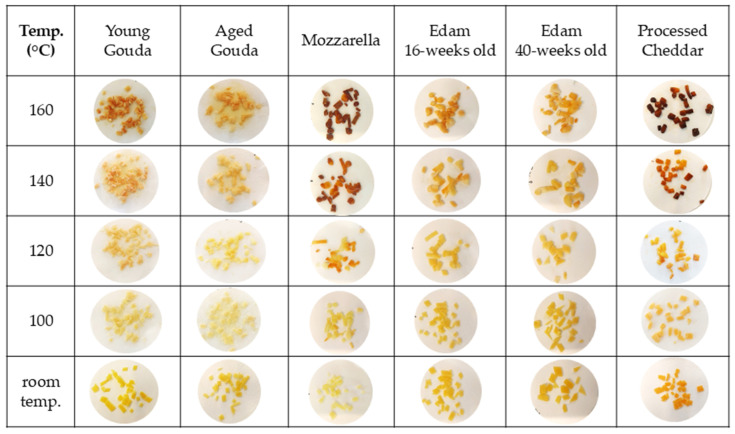
The effect of temperature on browning of pre-dried (24 h; 25 °C; 60% relative humidity) cheese (2 g) during 5 min of baking.

**Figure 10:**
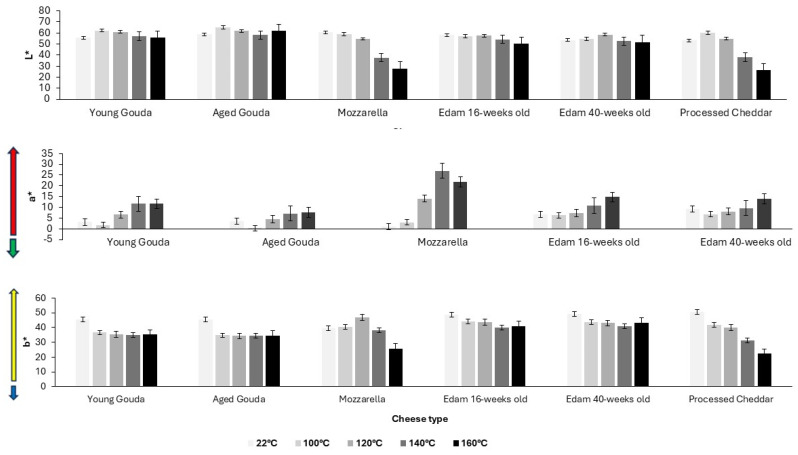
The effect of temperature on changes in color (CIELAB scale) of pre-dried cheese (2 g) after 5 min of baking. Vertical error bars represent one standard deviation.

As browning occurred only in samples after moisture loss had reached approx. 30%, six selected cheese samples were pre-dried in climate chamber (24 h; 25 °C; 60% relative humidity) to remove at least 30% of the moisture from cheese, and then a baking experiment was performed to investigate browning. The reduction in moisture content alone did not cause significant color change when compared to samples that did not undergo drying, indicating the importance of temperature in the browning effect. Lower moisture content limited the rate of moisture loss when compared to the baking experiment conducted with the cheeses at their initial moisture content (Figure 8). Pre-dried cheeses, which had a reduced initial moisture content, showed browning at lower temperatures (120 °C; Figure 9) than their counterparts which had not been pre-dried (Figure 4), and browning was less intensive as expressed by browning factor (Figure 6b). With a higher temperature of baking, the dominating colors were red and yellow for both fresh and pre-dried samples (Figure 5 and Figure 10).

For pre-dried cheese samples, the onset of browning was observed at lower temperatures compared to fresh samples (Figure 4 and Figure 9). This observation indicates that pre-drying reduces the moisture content, which accelerates the browning process during heating. In contrast, fresh samples, which contain higher moisture levels, require more time and higher temperatures for browning to initiate. Figure 4 and Figure 9 clearly illustrate this difference: pre-dried samples begin to exhibit browning at temperatures as low as 120 °C (young Gouda, processed Cheddar, and Mozzarella) to 140 °C (aged Gouda, and 16- and 40-weeks old Edam), while fresh samples show minimal or no browning until higher temperatures (140–200 °C) were applied. The extent and intensity of browning varied depending on cheese composition and aging stage. Furthermore, at 100 °C and 120 °C, the ΔE values were relatively low across all cheese types, with processed Cheddar showing the highest ΔE values of 13.7 and 12.4, respectively (Table 3). As the baking temperature increased to 140 and 160 °C, there was a marked increase in ΔE for most cheese types, indicative of intensified browning. Mozzarella exhibited the highest ΔE at 160 C, with a value of 41.1, followed by processed Cheddar at 39.1. In contrast, young and mature Gouda and Edam cheese (160 and 40-weeks old) displayed a lower color change during baking with a ΔE < 14, highlighting their reduced browning potential compared to other cheeses.

To assess the main factors affecting the rate of moisture loss, the mixed-effects model was used. In this model temperature of baking, the mass of cheese and time of baking were used as fixed factors. Replicates and initial moisture content were used as random factors. The results of the fixed effects tests (Table 4) showed that temperature had the most significant impact on moisture loss, as indicated by the highest F-value (2008.54) and *p*-value < 0.0001, followed by mass (F = 1973.28; *p* < 0.0001) and time (F = 278.49; *p* < 0.0001), indicating that all the variables included in the model had a significant effect on the moisture loss rate during cheese baking.

In a mixed-effects model, the random effects account for variability in the moisture loss rate that cannot be explained by the fixed effects alone. These factors are assumed to vary randomly and contribute to the overall variation. Two random factors—replicate and initial moisture content—were used in the model. The effect of initial moisture was statistically significant with *p*-value of 0.02. The effect of replicate was not significant (*p*-value > 0.05). The initial moisture content of the cheese accounted for 21.97% of the total variance in moisture loss rate (*p* = 0.02). A large portion of the variance (78.03%) of random factors was attributed to unexplained factors (model error) affecting moisture (e.g., cheese microstructure) that were not analyzed in this model.

The mixed-effects model explained 82.17% of the variance in the rate of moisture loss, which indicated a strong fit. The adjusted coefficient of determination value (82.09%) confirmed that the model accounted for most of the variation in the moisture loss rate. The standard deviation of the residual (S = 0.00057) indicated that, on average, the residuals deviated from the model’s predicted values by about 0.00057 units. The model coefficients summarized in Table 5 indicated the strength and direction of the relationship between each predictor variable and the moisture loss rate. Specifically, as the temperature increased from 100 to 200 °C, the moisture loss rate also increased.

The differences in moisture loss rate between the different temperatures were significant (Table 6). This was in agreement with the fact that higher temperatures promoted faster moisture evaporation. The effect of mass was also significant. The moisture loss rate increased with the increase in cheese mass. Smaller masses lost moisture at a slower rate than larger masses, as confirmed by a Tuckey post hoc test (Table 7). The effect of baking time was also significant, and the moisture loss rate increased as time progressed, peaking at 220 s (+0.000371 on average) (Table 5).

## 4. Discussion

This study analyzed the effects of cheese type, initial moisture content, mass, and baking temperature on moisture loss rates and browning characteristics. The findings demonstrated that baking conditions and inherent cheese properties significantly influence both moisture evaporation and browning, with implications for cheese quality in baked applications.

### 4.1. Moisture Loss Rate

The baking experiments showed that the process of moisture loss resembled the one observed during the spray drying of milk [32]. Initially, the drying rate (Figure 3, Figures S3 and S4) increased as the cheese reached the wet bulb temperature; this was followed by a steady rate, and finally, a reduction in moisture loss rate as moisture decreased, limiting further evaporation. The initial rapid loss corresponds to the evaporation of free water from the cheese surface, while the later phases reflect the progressive loss of bound water and structural changes that limit diffusion. Similarly to the results of this study, Schmitz-Schug et al. [33] observed two distinct stages during the spray drying of model dairy formulation. In the first stage, the drying rate remained constant, with a linear reduction in water content. In the second stage, drying became limited by heat conduction through the dry outer layer and the diffusion of water vapor from the core outward. Then, diffusivity decreased at low water content during this stage. Product temperature was close to the wet bulb temperature in the first stage but rose toward the air temperature in the second stage.

Temperature emerged as the most influential factor in the mixed-effects model (F = 2008.54, *p* < 0.0001), with higher baking temperatures significantly accelerating moisture loss (Table 4). This finding aligns with the data from the literature, which highlight the temperature-dependent nature of evaporation rates in high-moisture foods. Various models, including polynomial and exponential models, have been developed to describe drying kinetics across a wide range of food products [34,35], including dairy powders [33,36]. The higher the temperature of baking, the higher the moisture loss rate was observed in this study in the first stage of baking (Figure 3, Figures S3 and S4). By analogy to the spray drying of dairy products, temperature affects both the saturation pressure at the product surface and the diffusion coefficient. Elevated temperatures increase surface saturation pressure, enhancing evaporation during the first stage before crust formation [33]. As expected, higher temperatures (140–200 °C vs. 100–120 °C) resulted in faster moisture loss rates and lower final moisture contents during the second stage of baking in the current study (Figure 2, Figures S1 and S2). Higher temperatures improve diffusivity [37,38], facilitating mass transfer during the second stage, though diffusivity decreases at lower water content [33].

Cheese type and initial moisture content were also critical in determining moisture loss behavior in this study. High-moisture cheeses like Mozzarella and processed Cheddar showed faster rates of moisture loss than cheese varieties with a lower initial moisture content, such as aged Gouda and mature Cheddar (Figure 2 and Figure 3). This can most probably be attributed to the higher initial moisture content, which enhances water migration within the cheese matrix due to the greater availability of free water. By analogy, the results of this study could be compared with data related to the drying of other dairy products. The drying rates of skim milk, light milk, medium milk, whole milk, diluted coffee creamer, coffee creamer, and heavy cream have been studied [32,36] and analyzed as functions of water content (on a solid weight basis) and time at an air temperature of 70 °C. The findings also revealed that drying rates decreased with lower initial water content [36].

Cheese mass also influenced moisture loss in the current study (Figure 3, Figures S3 and S4), with larger masses (4–6 g) showing higher loss rates than smaller masses (2 g). However, an increase in moisture loss rates was less than proportional to the increase in cheese mass, which may be related to the moisture evaporation capacity of the system used. The airflow rate remained constant during baking, even though the baked cheese mass varied between 2 and 6 g. This likely led to a less-than-proportional increase in water loss when larger masses were baked. During baking, moisture from the semi-finished products increases the humidity inside the chamber. To prevent quality defects in the final products caused by high humidity, it must be removed through the vents [39].

The studied mixed-effects model revealed a strong correlation between baking temperature, initial moisture content, and moisture loss rates across different cheese types, with a R^2^ value of 82.17%, suggesting reliable predictive capacity. However, the model indicated that additional variables (78% of variability)—likely related to cheese microstructure, such as protein network arrangement and fat distribution [40]—may also play a significant role in moisture loss dynamics. Incorporating these structural factors into future models could improve predictive accuracy and deepen the understanding of water transport mechanisms in cheese during baking.

### 4.2. Browning

The onset of browning was observed when approximately 30% of moisture had been removed (Figure 7), with L* values declining more sharply at higher temperatures (Figure 5, Figures S7 and S8). The onset and intensity of browning were driven by temperature but modulated by cheese composition, particularly the initial moisture content and ripening stage (Figure 6 and Figure S9). As illustrated in Figure 4, browning began at lower temperatures (140 °C) for Mozzarella (46.6% moisture; 41% FDM; 0.08% lactose and 0.67% galactose; Table 1) and processed Cheddar (56% moisture; 38% FDM; 6% sugar; Table 1), compared to 160 °C or higher for aged cheeses like mature Cheddar (36.3% moisture; 54% FDM; <0.2% sugar) or aged Gouda (moisture; 55% FDM; 0% sugar; Table 1). The onset of browning at lower temperatures in high-moisture cheeses can be attributed to localized drying, which concentrates reactants like lactose or residual sugars at the surface, thereby facilitating Maillard reactions. Residual sugar content in cheese can constitute up to 6% of the total mass, depending on the cheese variety and the extent of fermentation [11,41], as observed in the processed Cheddar and Mozzarella used in this study (product label). Starter cultures used in cheese production metabolize lactose and generate galactose as a byproduct [42]. While some starter cultures can ferment galactose, others cannot, leading to its accumulation in cheese. The thermophilic cultures typically used for production of Mozzarella cannot ferment galactose [43]. This presence of galactose can contribute to enhanced browning during processing [17,44]. A positive correlation between galactose content and brown color intensity has been reported for Mozzarella when subjected to heating [41]. The Maillard reaction in processed cheese is accelerated by high pH levels in the final product (>5.9) and storage at elevated temperatures (35 °C) [45,46]. The properties of the blend used for manufacturing will also alter the extent of browning in processed cheese. Specifically, high levels of residual galactose and lactose in Cheddar cheese, especially when combined with a high salt-in-moisture content, lead to more pronounced browning in the final processed product [47].

It is also worth noting that cheeses with higher fat content (mature Cheddar, Gouda) browned at higher temperatures compared to cheeses with lower fat content like Mozzarella and processed Cheddar (Table 1). As reported by Ma et al. [5], for cheese containing higher free oil, like Gruyere and Provolone, a sufficient amount of free oil covers vapor bubbles, which prevents moisture evaporating from cheese; hence, less intensive browning occurs. In contrast, Mozzarella has much less free oil covering the bubbles, from which the moisture in cheese evaporates more easily, leaving a burnt surface on each blister [5].

Additionally, as noted by Lee et al. [11], the lactose and galactose content in cheese may lead to an overestimation of moisture when a microwave-based method is used for moisture analysis. These sugars can react with other compounds in the cheese under the thermal conditions required for measurement, producing volatile reaction products (quantitatively, 1 mol of lactose reacted with equimolar lysine can yield up to 1 mol of maltol and 2 mol of water). The study by Lee et al. [11] demonstrated that residual sugars can cause moisture determinations to be overestimated by values of up to 1.8. In this study, Mozzarella and processed Cheddar were characterized by the highest ratio of water loss; however, in the current study, a halogen moisture analyzer was employed, so the mechanism of evaporation might have not been the same.

The role of ripening in browning intensity is particularly noteworthy. Ripened cheeses contain elevated levels of free amino acids and peptides formed through proteolysis, which are key substrates for Maillard reactions [48,49]. These precursors accelerate browning, as evidenced by the pronounced redness (a* values) in mature Cheddar and aged Gouda in the current study (Figure 5).

### 4.3. Comparison of Fresh and Pre-Dried Cheeses

The comparison of the change in color of fresh and pre-dried cheeses (Table 2 and Table 3, Figure 4, Figure 6 and Figure 9) after baking further emphasized the critical role of moisture reduction in enhancing browning reactions. Pre-dried samples started browning at lower baking temperatures compared to fresh samples likely due to decreased water content, which concentrates reactants (e.g., lactose and free amino acids) on the cheese surface. The substantial ΔE values observed for Mozzarella and processed Cheddar at 160 °C can be attributed to their high carbohydrate content (Table 3). Mozzarella, with its high lactose content, and processed Cheddar, often fortified with added sugars, provide abundant substrates for the Maillard reaction and caramelization [17,47]. The moderately high ΔE values for Edam (16 weeks old) and aged Gouda (Table 3) reflect the accumulation of free amino acids during ripening, which enhances browning potential when moisture is reduced [3,48]. The higher value for young Gouda may be connected with its less pronounced red color at the beginning of ripening when compared to more mature ones (Figure 10).

When comparing browning at 200 °C (when all cheeses have undergone an extensive change in color) between fresh and pre-dried samples, most of the pre-dried cheeses, except processed Cheddar, were characterized by a slightly smaller change in color with a lower presence of red (Figure 4 and Figure 9). The visual observations were confirmed by quantitative analysis, with the same relation between ΔE values and a* values (Figure 5 and Figure 10, Table 2 and Table 3). The Maillard reaction is moisture dependent on optimum water activity. It can be inhibited by reducing the moisture content through dehydrating procedures [14].

The profile of moisture loss rate for pre-dried samples (Figure 8) was different when compared to their fresh counterparts (Figure 3). The moisture loss rate was lower for pre-dried samples, and the so-called stable rate state (second stage) dominated over the course of baking. By analogy to the spray-drying process of dairy products [36], the presence of a single drying period could be attributed to the lower initial water content in cheese.

## 5. Conclusions

This study demonstrated the critical influence of cheese type, initial moisture content, mass, and baking temperature on moisture loss kinetics and browning behavior during baking. The moisture loss kinetics mirrored typical spray-drying patterns, with rapid initial evaporation followed by a slower, diffusion-limited phase. As revealed by the analysis of the results of the mixed-effect model, temperature was the dominant factor, significantly accelerating water loss, followed by cheese mass. Initial moisture content explained 22% of the variability in this model, suggesting the contribution of other factors, such as fat distribution, casein network structure, porosity, and surface exposure during baking, which could provide a more comprehensive understanding of the moisture loss and browning process in future studies. Further investigations into these aspects are recommended for future study. High-moisture cheeses like Mozzarella exhibited faster initial moisture loss due to greater water availability, while aged cheeses (Gouda, Edam, and Cheddar), with lower moisture contents, showed slower rates. Higher baking temperatures, particularly above 140 °C, accelerated moisture evaporation. All tested cheeses required a mass reduction of approximately 30%—assuming that only water evaporated during baking—for browning to initiate, regardless of the cheese type. Lowering the moisture content in cheese prior to baking resulted in a less intense brown color after baking. The interplay between cheese ripening and baking outcomes underscores the critical role of cheese composition in both culinary and industrial applications. Ripened cheeses, such as mature and aged Gouda and mature Cheddar, with their lower moisture content and higher levels of Maillard reaction precursors, are particularly suitable for applications that require intense browning at higher baking temperatures—such as pizza toppings. These cheeses begin to brown at higher temperatures compared to Mozzarella, with optimal browning occurring at around 160 °C for Gouda and Cheddar, compared to Mozzarella’s 140 °C. However, Mozzarella and processed Cheddar exhibited exceptional browning properties among all the tested cheeses. Brown spots appeared at the lowest baking temperatures, and their color deepened more intensely compared to other cheeses, likely due to their higher carbohydrate content (such as lactose and galactose).

These findings have practical implications for optimizing baking conditions. Adjusting factors such as temperature and cheese mass based on cheese type can help to achieve the desired visual outcomes while minimizing issues like excessive drying or uneven browning. While the experimental setup used in this study provided notable fundamental insights, future studies should also expand into conditions typically used for baking, e.g., the baking of pizzas and pasta dishes containing cheese, considering aspects like oven type, air circulation, and other factors. Additionally, future studies could expand on the assessment of browning by sensory evaluations or flavor profiling to better understand the sensory impact of browning. Furthermore, a broader range of cheeses with diverse compositions, such as Parmesan or Brie, could help to enhance the generalizability of the results, offering a more comprehensive understanding of how different cheeses behave under thermal processing and allowing translation to practical guidelines for cheese baking.

## Figures and Tables

**Table 1:** Chemical composition of tested cheese types.

Cheese Type	Chemical Composition		
	FDM (%)	Salt in Dry Matter (%)	Moisture * (%)
Mozzarella (4 wk)	41	2.2	46.6 ^b^ ± 0.4
Edam (16 wk)	43	3.6	45.9 ^b,c^ ± 0.3
Edam (40 wk)	43	3.6	36.0 ^d^ ± 0.1
Young Gouda (4 wk)	55	3.4	44.5 ^c^ ± 0.5
Mature Gouda (8 wk)	55	3.4	43.3 ^c^ ± 0.2
Aged Gouda (52 wk)	55	3.4	35.7 ^d^ ± 0.3
Mature Cheddar (52 wk)	54	3.2	36.3 ^d^ ± 0.1
Processed Cheddar	38	8.6	56.0 ^a^ ± 0.1

FDM—fat in dry matter; * moisture content in cheese was measured using the oven drying method as described in Section 2.2.; ^a–d^—values in the column not sharing a common superscript are significantly different (*p* < 0.05); wk—weeks old.

**Table 2:** Total color change (ΔE) in fresh cheese samples baked at temperature range 100–200 °C, calculated according to Esteller et al. [31].

Temperature of Baking (°C)	YG	MG	AG	M	E16	E40	PC	MC
100	3.5 ± 0.3	0.5 ± 0.1	3.1 ± 0.1	2.3 ± 0.1	36.4 ± 0.1	4.3 ± 0.1	22.0 ± 1.1	0.2 ± 0.0
120	2.5 ± 0.1	12.6 ± 0.4	13.8 ± 0.3	2.2 ± 0.1	28.7 ± 0.7	1.8 ± 0.1	19.3 ± 0.8	0.8 ± 0.0
140	2.5 ± 0.1	10.9 ± 0.4	12.0 ± 0.3	3.8 ± 0.1	38.7 ± 0.1	4.7 ± 0.4	17.7 ± 0.8	2.0 ± 0.1
160	7.7 ± 0.6	3.7 ± 0.3	5.8 ± 0.3	15.3 ± 1.6	39.2 ± 0.1	10.6 ± 1.1	20.9 ± 0.9	14.5 ± 0.7
180	19.4 ± 0.6	20.5 ± 2.3	22.5 ± 2.2	38.8 ± 4.5	47.5 ± 2.1	22.0 ± 1.5	22.7 ± 2.3	16.7 ± 0.6
200	32.4 ± 0.8	25.2 ± 4.5	27.1 ± 4.3	49.9 ± 4.3	50.4 ± 2.7	28.8 ± 3.5	36.9 ± 2.1	21.4 ± 0.6

Results are expressed as mean ± standard deviation; YG—young Gouda; MG—mature Gouda; AG—aged Gouda; M—Mozzarella; E16—Edam 16-weeks old; E40—Edam 40-weeks old; PC—processed Cheddar; MC—mature Cheddar.

**Table 3:** Total color change (ΔE) in pre-dried cheese samples baked at temperature range 100–200 °C, calculated according to Esteller et al. [3].

Temperature of Baking (°C)	YG	AG	M	E16	E40	PC
100	11.2 ± 0.9	12.8 ± 0.1	2.8 ± 0.1	4.9 ± 0.2	6.4 ± 0.4	13.7 ± 0.3
120	11.8 ± 0.7	11.7 ± 0.5	16.1 ± 0.2	5.2 ± 0.3	8.0 ± 0.3	12.4 ± 0.7
140	13.5 ± 0.8	11.7 ± 0.7	34.6 ± 3.2	10.5 ± 0.1	8.4 ± 0.6	24.6 ± 1.5
160	13.3 ± 0.8	12.3 ± 0.5	41.1 ± 2.0	14.0 ± 0.4	7.8 ± 0.4	39.1 ± 3.8

Results are expressed as mean ± standard deviation; YG—young Gouda; AG—aged Gouda; M—Mozzarella; E16—Edam 16-weeks old; E40—Edam 40-weeks old; PC—processed Cheddar.

**Table 4:** Tests of fixed effects calculated with mixed-effects model while modeling the moisture loss rate (g/s) during cheese baking.

Factor	DF Number	DF Den	F-Value	*p*-Value
Temperature of baking (°C)	5.00	4290.89	2008.54	<0.0001
Mass of cheese (g)	2.00	4294.47	1973.28	<0.0001
Time of baking (s)	14.00	4288.64	278.49	<0.0001

DF—degrees of freedom.

**Table 5:** Coefficients calculated in mixed-effects model.

Term	Coefficient	SE	DF	T-Value	*p*-Value
Constant	0.002538	0.000096	8.64	26.36	<0.0001
Temp. (°C)					
100	−0.001178	0.000020	4291.77	−60.41	<0.0001
120	−0.000856	0.000020	4291.77	−43.90	<0.0001
140	−0.000333	0.000019	4290.07	−17.15	<0.0001
160	0.000237	0.000019	4289.63	12.20	<0.0001
180	0.000794	0.000019	4291.67	40.75	<0.0001
Mass (g)					
2	−0.000755	0.000012	4295.21	−60.70	<0.0001
4	0.000164	0.000012	4295.21	13.20	<0.0001
Time (s)					
20	−0.001577	0.000032	4288.63	−48.62	<0.0001
40	−0.000835	0.000032	4288.63	−25.73	<0.0001
60	−0.000440	0.000032	4288.63	−13.56	<0.0001
80	−0.000198	0.000032	4288.63	−6.11	<0.0001
100	−0.000001	0.000032	4288.63	−0.04	0.966
120	0.000136	0.000032	4288.63	4.18	<0.0001
140	0.000237	0.000032	4288.63	7.32	<0.0001
160	0.000299	0.000032	4288.63	9.21	<0.0001
180	0.000338	0.000032	4288.63	10.44	<0.0001
200	0.000366	0.000032	4288.63	11.29	<0.0001
220	0.000371	0.000032	4288.63	11.43	<0.0001
240	0.000361	0.000032	4288.65	11.14	<0.0001
260	0.000344	0.000032	4288.65	10.60	<0.0001
280	0.000318	0.000032	4288.65	9.82	<0.0001

SE—standard error; DF—degrees of freedom.

**Table 6:** Differences between mean moisture loss rate depending on the baking temperature (the Tukey post hoc test and 95% confidence level).

Baking Temperature (°C)	N	Mean Moisture Loss Rate (g/s)	Grouping					
200	720	0.0039	A					
180	720	0.0033		B				
160	720	0.0028			C			
140	720	0.0022				D		
120	720	0.0017					E	
100	720	0.0014						F

Means that do not share a letter are significantly different; N—sample size.

**Table 7:** Differences between mean moisture loss rate depending on the mass of cheese baked (the Tukey post hoc test and 95% confidence level).

Cheese Mass (g)	N	Mean Moisture Loss Rate (g/s)	Grouping		
6	1440	0.0031	A		
4	1440	0.0027		B	
2	1440	0.0018			C

Means that do not share a letter are significantly different; N—sample size.

## Data Availability

The original contributions presented in this study are included in the article/Supplementary Materials. Further inquiries can be directed to the corresponding author.

## Supplementary Materials

The following supporting information can be downloaded at https://www.mdpi.com/article/10.3390/foods14020165/s1. Figure S1: The effect of baking temperature and initial moisture content of cheese on moisture content during baking over time of cheese (4 g) baking; Figure S2: The effect of baking temperature and initial moisture content of cheese on moisture content during baking over time of cheese (6 g) baking; Figure S3: The effect of temperature and initial moisture content of cheese on the change in moisture loss over time of cheese (4 g) baking; Figure S4: The effect of temperature and initial moisture content of cheese on the change in moisture loss over time of cheese (6 g) baking.; Figure S5: The effect of temperature on cheese (4 g) browning after 5 min of baking; Figure S6: The effect of temperature on cheese (6 g) browning after 5 min of baking; Figure S7: The effect of temperature on changes in color (CIELAB scale) of different types of cheese (4 g) after 5 min of baking; Figure S8: The effect of temperature on changes in color (CIELAB scale) of different types of cheese (6 g) after 5 min of baking; Figure S9: The browning factor calculated for fresh cheese samples (4 and 6 g) after 5 min of baking.
