# Supplementary material for: Moisture Loss from Cheese During Baking: Influence of Cheese Type, Cheese Mass, and Temperature
Source: Foods. 2025 Jan 8;14(2):165. doi: 10.3390/foods14020165 (PMC11764551; doi:10.3390/foods14020165)
Supplement: Supplementary file 1 [file foods-14-00165-s001.zip › foods-3371577-supplementary.pdf]

Supplementary material

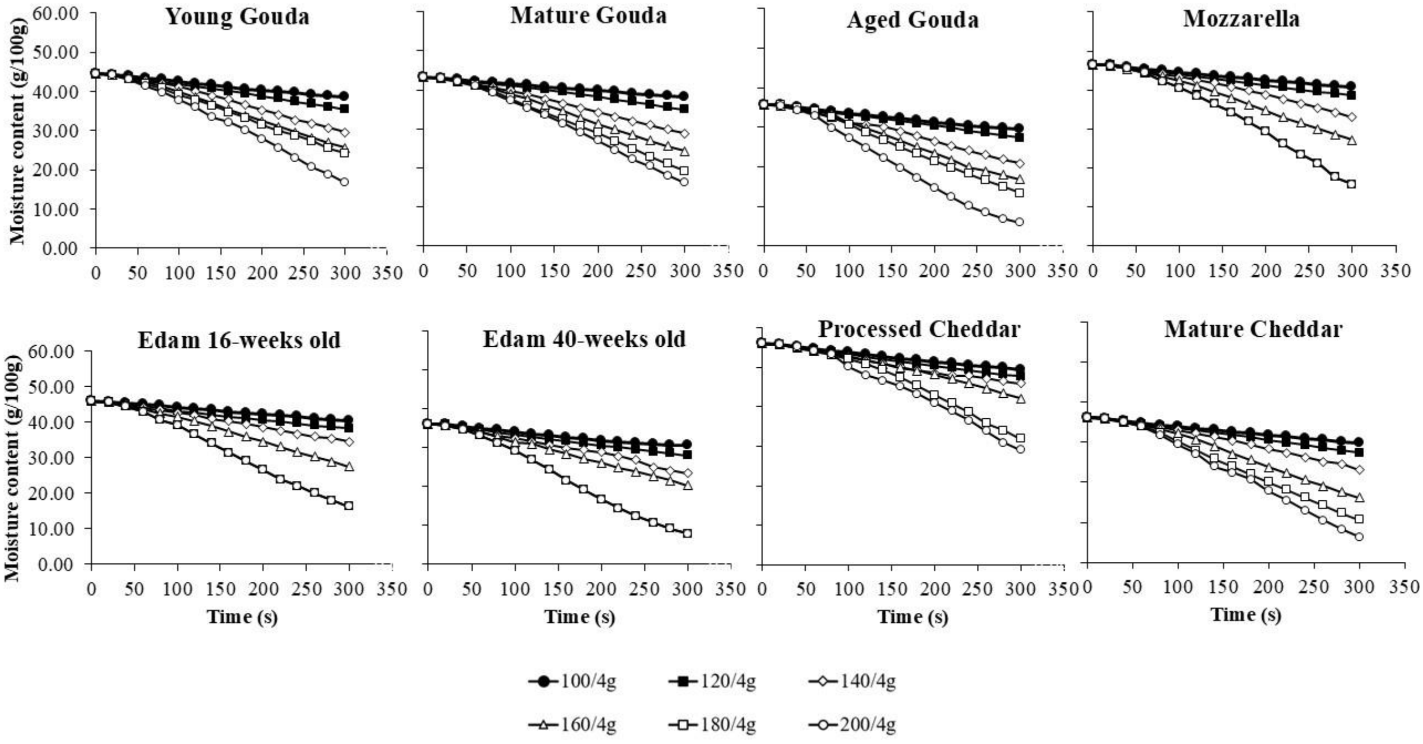

Figure S1. The effect of baking temperature and initial moisture content of cheese on moisture content during baking over time of cheese (4 g) baking.

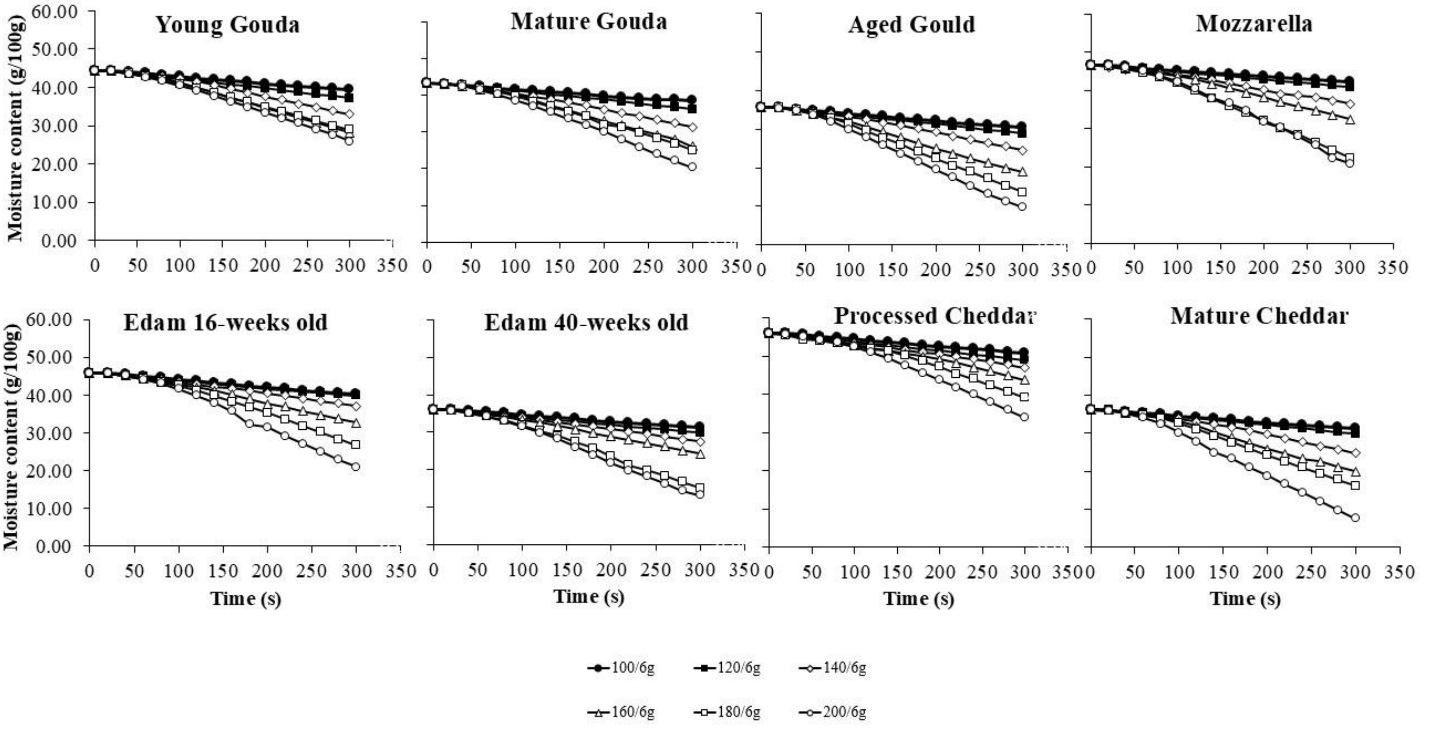

Figure S2. The effect of baking temperature and initial moisture content of cheese on moisture content during baking over time of cheese (6 g) baking.

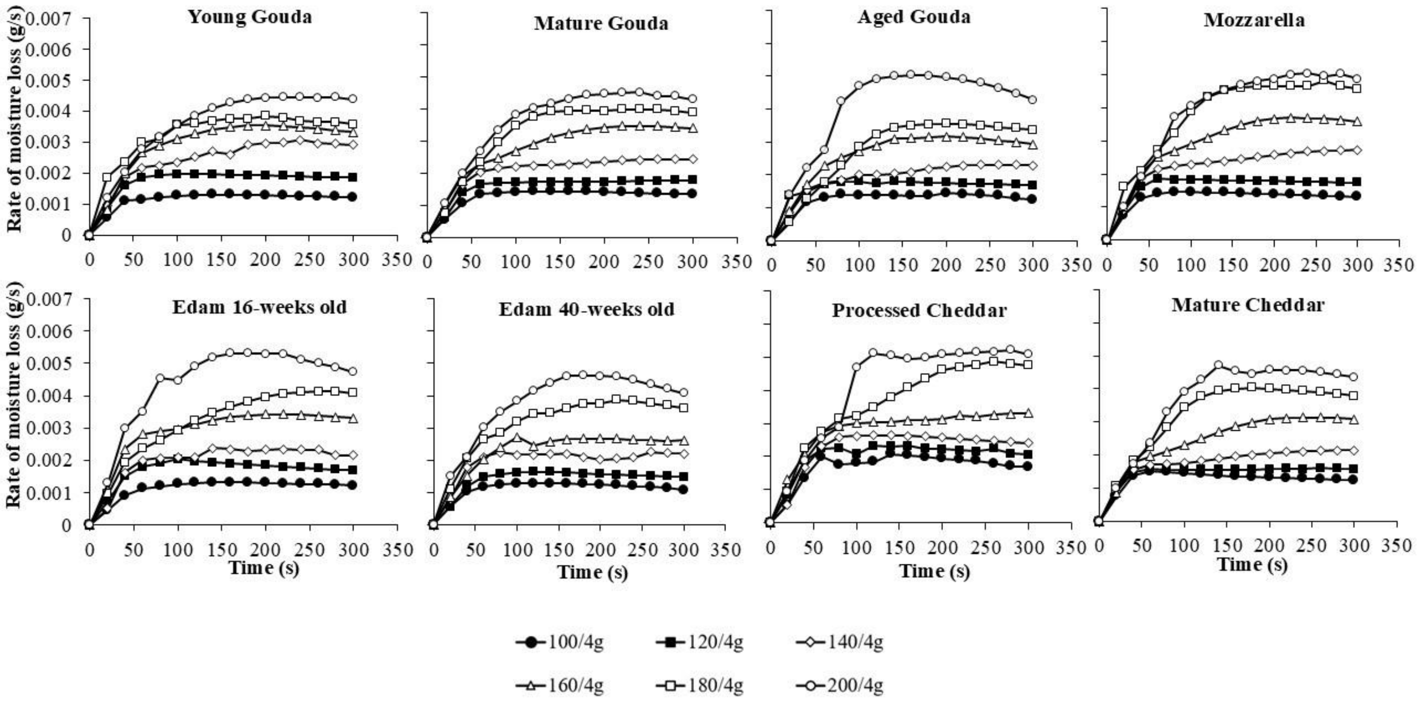

Figure S3. The effect of temperature and initial moisture content of cheese on the change in moisture loss over time of cheese (4 g) baking.

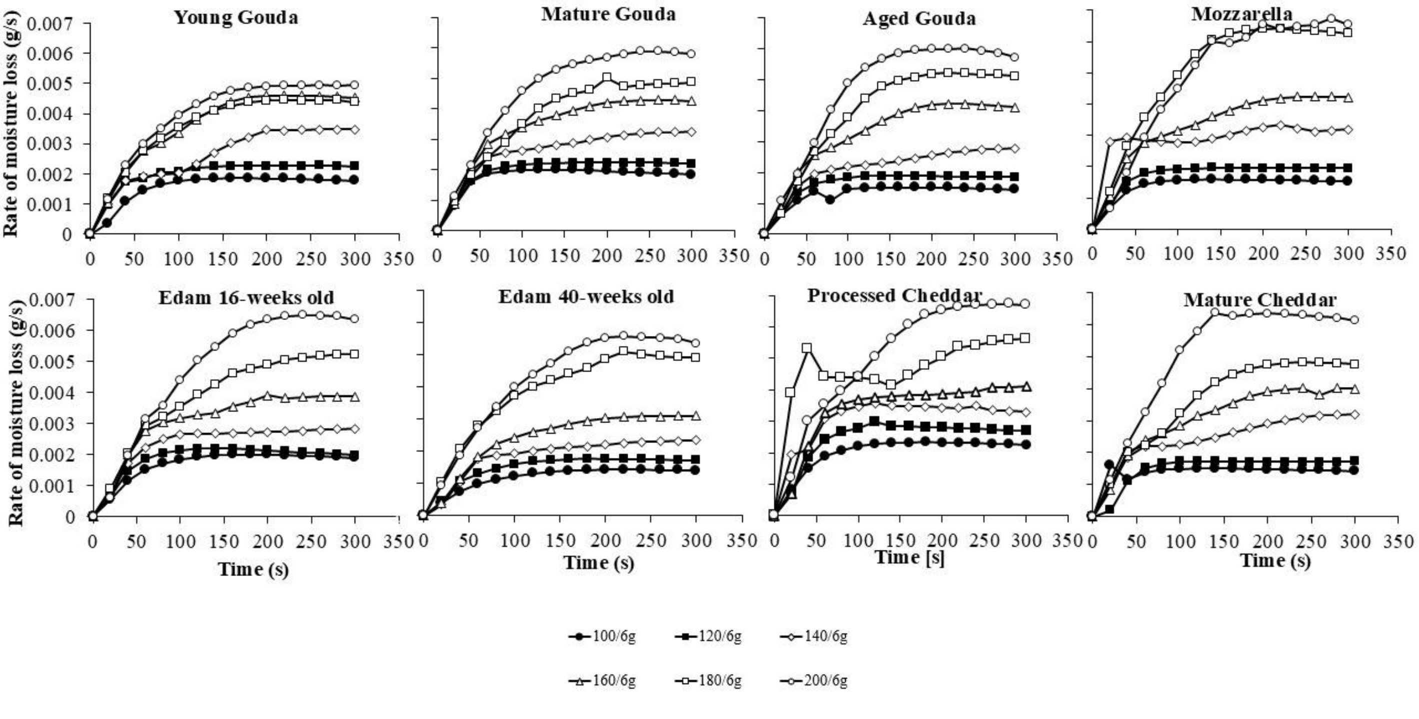

Figure S4. The effect of temperature and initial moisture content of cheese on the change in moisture loss over time of cheese (6 g) baking.

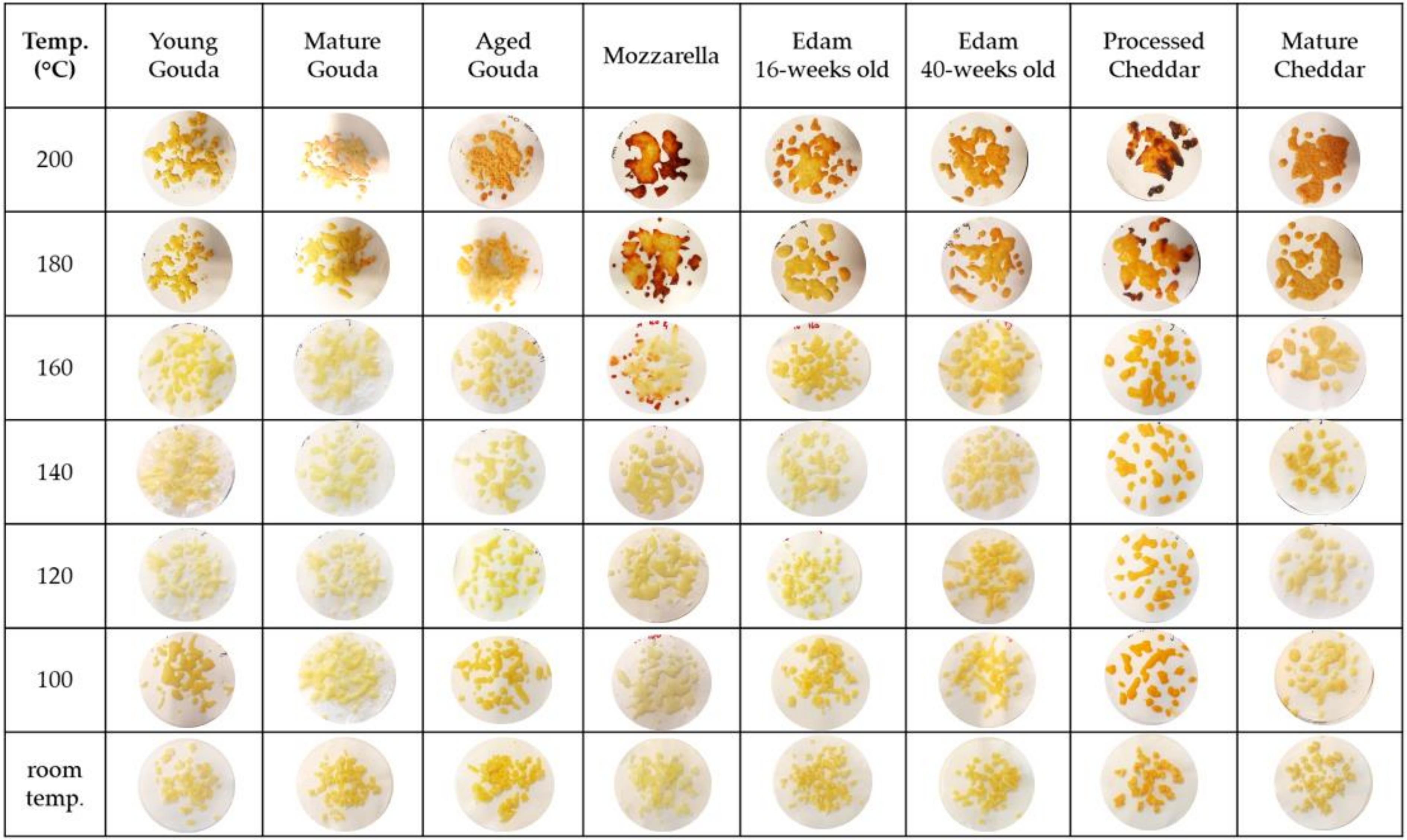

Figure S5. The effect of temperature on cheese (4 g) browning after 5 min of baking.

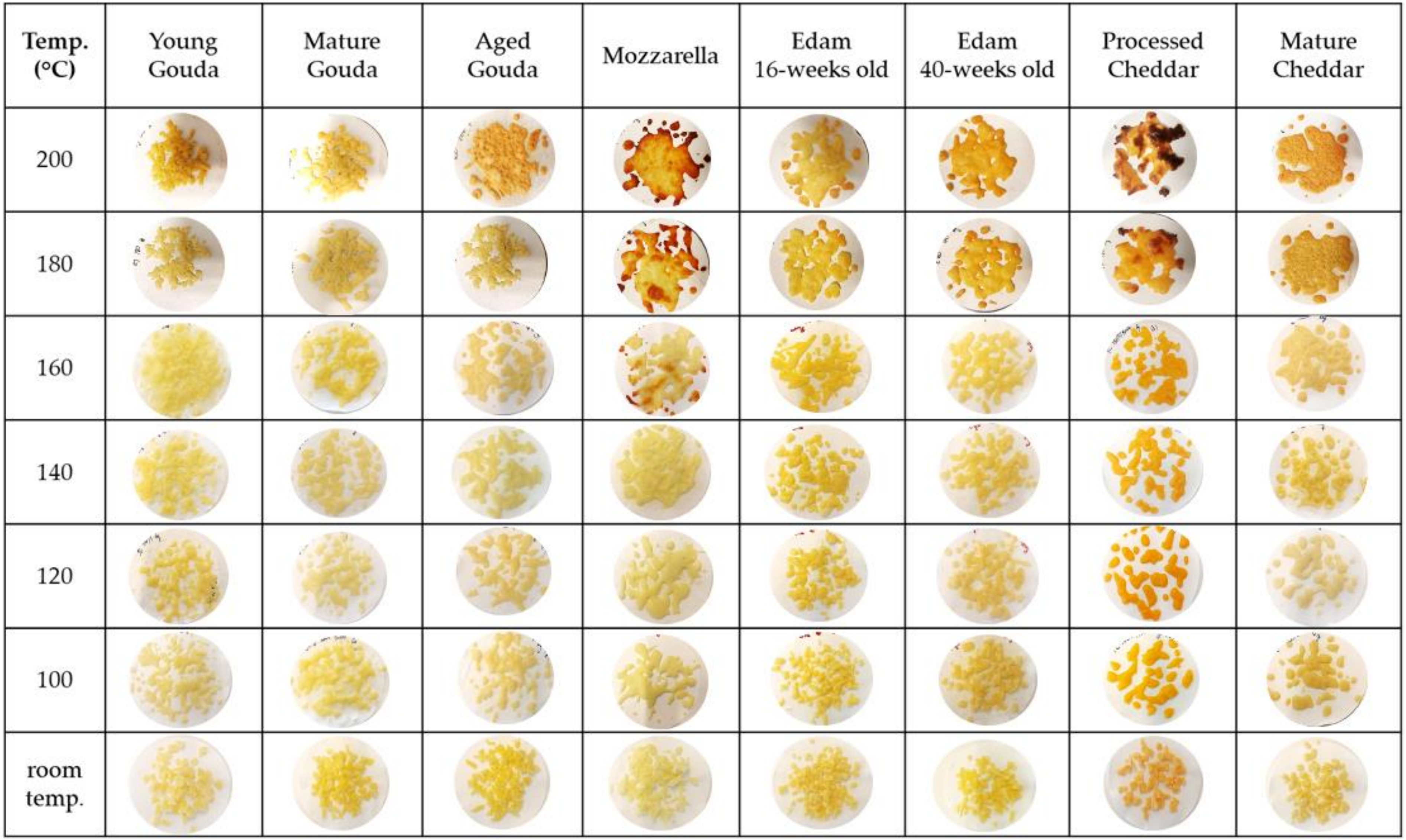

Figure S6. The effect of temperature on cheese (6 g) browning after 5 min of baking.

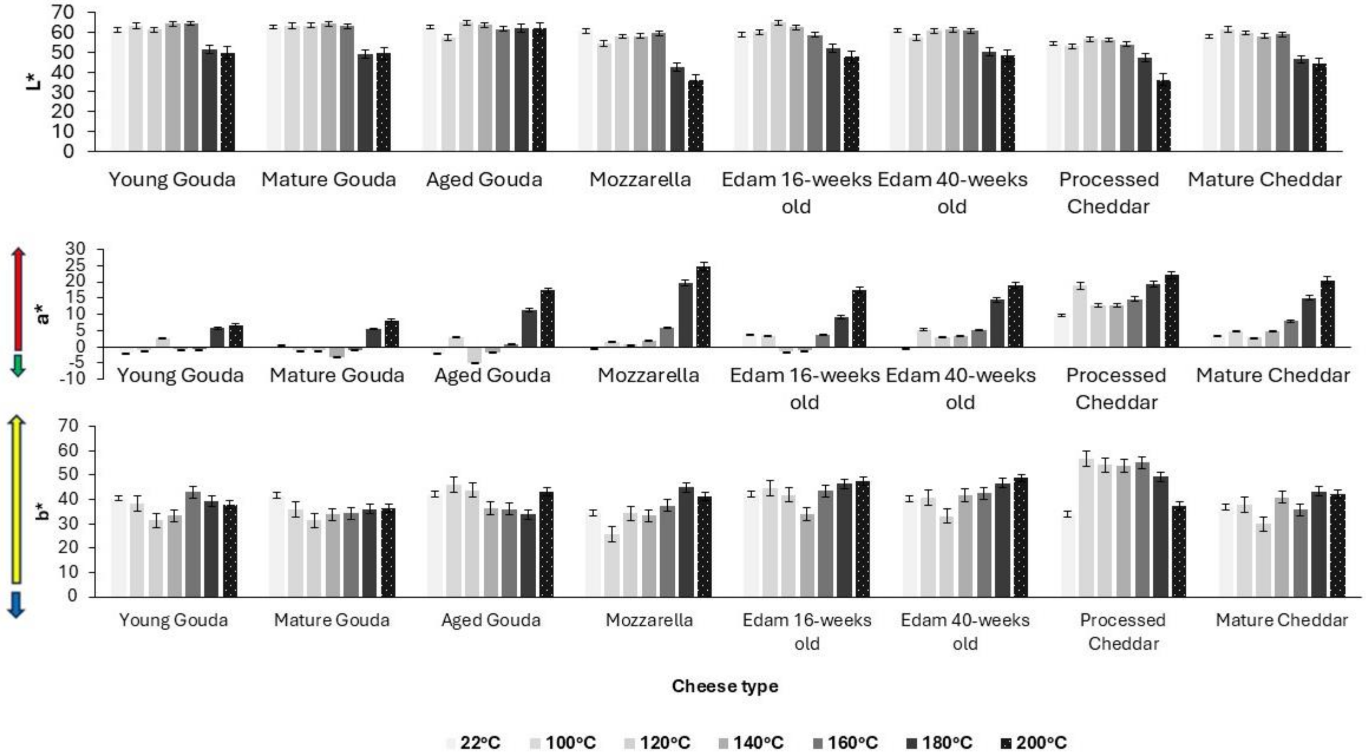

Figure S7. The effect of temperature on changes in color (CIELAB scale) of different type of cheese (4 g) after 5 min of baking. Vertical error bars represent one standard deviation.

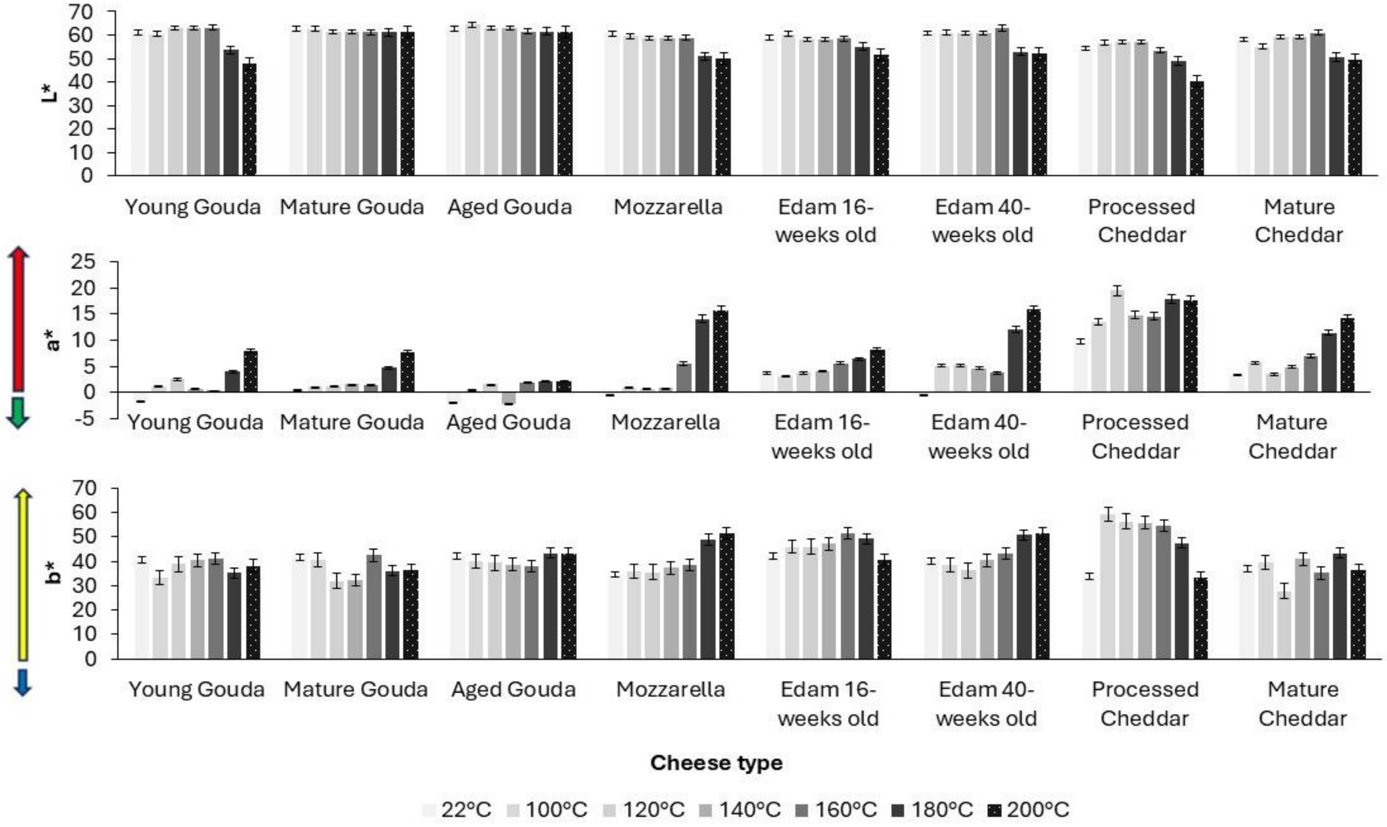

Figure S8. The effect of temperature on changes in color (CIELAB scale) of different type of cheese (6 g) after 5 min of baking. Vertical error bars represent one standard deviation.

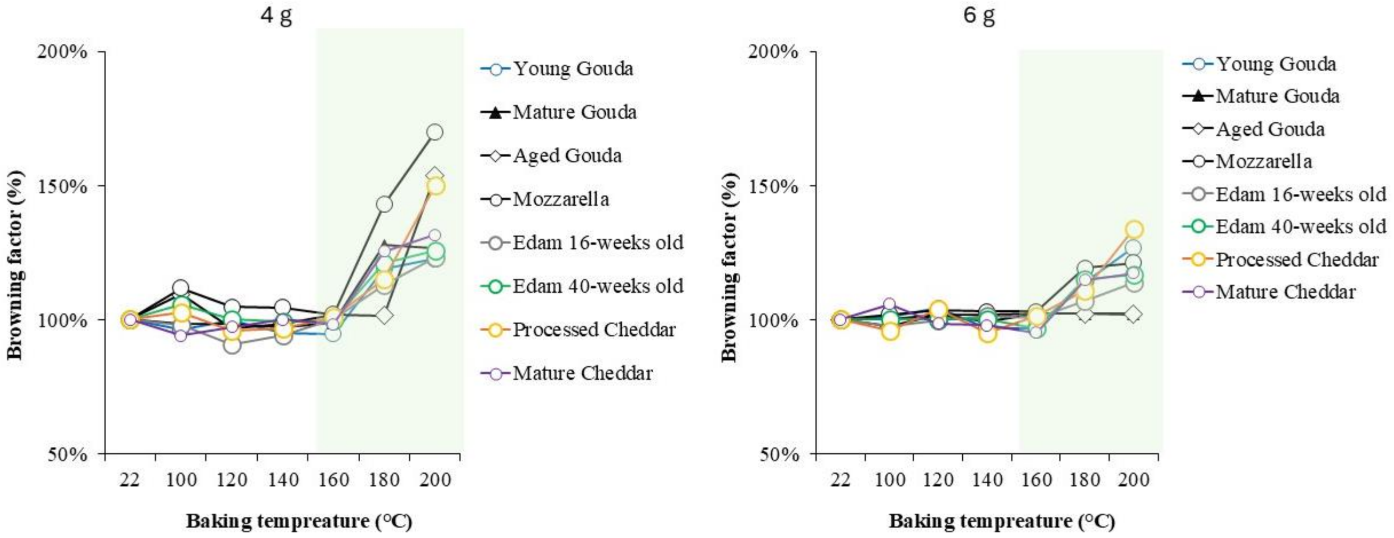

Figure S9. The browning factor calculated for fresh cheese samples (4 and 6 g) after 5 min of baking.
